# Chronic Progressive Neurodegeneration in a Transgenic Mouse Model of Prion Disease

**DOI:** 10.3389/fnins.2016.00510

**Published:** 2016-11-11

**Authors:** Nina Fainstein, Dvir Dori, Kati Frid, Alexa T. Fritz, Ilona Shapiro, Ruth Gabizon, Tamir Ben-Hur

**Affiliations:** Department of Neurology, The Agnes Ginges Center for Human Neurogenetics, Hadassah – Hebrew University Medical CenterJerusalem, Israel

**Keywords:** neurodegeneration, animal model, aging, prion diseases, neurogenesis

## Abstract

Neurodegenerative diseases present pathologically with progressive structural destruction of neurons and accumulation of mis-folded proteins specific for each condition leading to brain atrophy and functional disability. Many animal models exert deposition of pathogenic proteins without an accompanying neurodegeneration pattern. The lack of a comprehensive model hinders efforts to develop treatment. We performed longitudinal quantification of cellular, neuronal and synaptic density, as well as of neurogenesis in brains of mice mimicking for genetic Creutzfeldt-Jacob disease as compared to age-matched wild-type mice. Mice exhibited a neurodegenerative process of progressive reduction in cortical neurons and synapses starting at age of 4–6 months, in accord with neurologic disability. This was accompanied by significant decrease in subventricular/subependymal zone neurogenesis. Although increased hippocampal neurogenesis was detected in mice, a neurodegenerative process of CA1 and CA3 regions associated with impaired hippocampal-dependent memory function was observed. In conclusion, mice exhibit pathological neurodegeneration concomitant with neurological disease progression, indicating these mice can serve as a model for neurodegenerative diseases.

## Introduction

Neurodegenerative diseases are late-onset fatal disorders that affect large numbers of individuals (Hamacher and Marcus, 2007). In addition to the progressive death of neurons, neurodegenerative diseases cause accumulation of aberrantly-folded “key” disease proteins (Morales and Green, 2009; Aguzzi and O'Connor, 2010; Perry et al., 2012), which individually characterize each of these conditions, including amyloid β in Alzheimer's disease (AD), α synuclein in Parkinson's disease (PD) and prion protein (PrP^Sc^) in prion diseases such as Creutzfeldt-Jakob disease (CJD) (Prusiner, 1982; Olanow and Prusiner, 2009). These aggregates are considered hallmarks of neurodegenerative diseases (Ashe and Aguzzi, 2013; Prusiner, 2013). The mechanisms which result in abnormal proteins deposition and neurodegeneration are not entirely clear. Several common mechanisms of injury have been described in various neurodegenerative diseases, including prion diseases. These include the toxic effects of misfolded proteins (Doyle et al., 2011), oxidative stress and lipid and protein oxidation (Grimm et al., 2011), microglial activation (Heneka et al., 2014), activation of autophagy by intracellular protein aggregates (Yang et al., 2013), and dysfunction of other cellular functions, such as proteasome and mitochondria (Figueiredo-Pereira et al., 2014).

The animal models which were constructed for these conditions demonstrate accumulation of aberrantly-folded proteins. However, they are devoid of other pathological signs of neurodegeneration such as progressive neuronal loss and severe clinical features. In this project, we studied the presence of the neurodegenerative process in a mouse model of genetic CJD.

## TgMHu2ME199K/KO: a transgenic mouse model of familial prion disease

Prion diseases are a group of neurodegenerative diseases associated with deposition of abnormal prion protein in the brain. Pathologically, there is neuronal loss and atrophy in the cortex and deep-seated nuclei, which is associated with diffuse and plaque-like deposition of PrP, and accumulation of brain vacuoles surrounded by PrP deposits(Puoti et al., 1999; Gambetti et al., 2003). The most common genetic form of the disease (familial Creutzfeldt-Jakob disease, fCJD) is caused by a point mutation in codon 200 of the PrP gene, replacing Lysine (K) for glutamate (E) (Hsiao et al., 1991). The murine PrP gene differs from its human counterpart by 28 amino acids. A human / murine chimeric gene has been constructed which is mostly murine, but contains nine amino acids from the human gene between codons 96 and 167 (Telling et al., 1994). Unlike Tg mice expressing the human PrP gene, animals expressing chimeric mouse human PrP can be easily infected with human prions (Telling et al., 1995). Insertion of the E to K mutation at the relevant position (codon 199, representing the human E200K mutation) led to the generation of a transgenic mouse line (TgMHu2ME199K/KO). These mice suffer from progressive neurological symptoms as early as 5–6 month of age and deteriorate to a terminal condition several months thereafter, concomitant with the accumulation of a truncated form of protein kinase-resistant PrP (Kovacs et al., 2010; Friedman-Levi et al., 2011, 2013). TgMHu2ME199K/KO mice exhibit typical pathological features of human CJD, such as gliosis and lipid oxidation, and have already been used successfully to test the activity of a candidate reagent (Mizrahi et al., 2014)

In this project, we characterized the neurodegenerative pathological features of TgMHu2ME199K/KO mice in relation to age and clinical disease progression. We found a facilitated progressive reduction in cortical neurons and synapses as compared to normal aging, starting at age 4–6 months, according with neurologic disability. There was significant decrease in neurogenesis in the subventricular / subependymal zone. A similar progressive loss of neurons in the CA1 and CA3 regions of the hippocampus was associated with impaired hippocampal-dependent memory functions. Interestingly, there was increased hippocampal neurogenesis in TgMHu2ME199K/KO mice prior to decline in memory functions. Therefore, we believe the TgMHu2ME199K/KO model can serve both to study the neurodegenerative pathways as well as to test neuroprotective drugs and regenerative therapies that may be relevant to this and other neurodegenerative conditions.

## Methods

### Animals

Twenty-five C57BL/6 mice and 23 TgMHu2ME199K/KO mice of murine PrP^C^ knockout C57BL/6 mouse background (Friedman-Levi et al., 2011) were used in this study. All mice were grown under Specific-pathogen free conditions. Animal experimentation was approved by the institutional ethics committee.

### Clinical scoring and evaluation of hippocampus-dependent memory

Mice were scored for disease severity and progression according to a scale of clinical signs designed by us to fit the clinical symptoms observed (Friedman-Levi et al., 2011) in the Tg mice. Hind limb mild weakness = 1, Hind limb partial paralysis = 2, Full paralysis in one limb = 3, Full hind limb paralysis = 4, Death = 5. Hind limb weakness was first evaluated by closely watching the mouse walking on a flat surface, looking for signs of abnormal limb posture or abnormal walking pattern (high or low gait, leg-dragging). Next, mice were tested for their ability to walk in a straight line on a 3 cm beam and maintain balance. Finally mice were lifted by their tail to check for leg-clasping. Full paralysis constituted total lack of movement in the limb. This scale of scoring was proven to be parallel to the NSS (neurological severity score). Follow-up was performed blindly on a daily basis by two separate observers. This type of follow up allows tracking the decline of motor performance of animals in a precise manner.

The object recognition test (Bevins and Besheer, 2006) was used as a measure for hippocampal dependent memory. The test was carried out on 5–7 wt mice and 4–7 TgMHu2ME199K/KO mice at each time point (ages 2, 4, 6, and 10 months). Data are presented as fraction of time the animal spent actively exploring the new object from total time of object exploration.

### Histopathology

At each time point (ages 2, 4, 6, and 10 months), 5–7 mice per group (WT and TgMHu2ME199K/KO) were sacrificed for histopathological evaluation. Mice were anesthetized with a lethal dose of pentobarbital and brains were perfused via the ascending aorta with ice-cold PBS followed by cold 4% paraformaldehyde. Tissues were deep-frozen in liquid nitrogen. Serial 10 μM coronal sections were made. Immunofluorescent staining for mouse anti-NeuN (Millipore) and rabbit anti-synaptophysin (Cell-Marque) were performed, followed by incubation with Alexa-fluor555 goat anti-mouse IgG and Alexa-fluor555 goat anti-rabbit IgG respectively, as previously described (Fainstein et al., 2013). Nuclear counterstain was performed using DAPI (Vectashield). Analysis was performed on each hemisphere in four brain sections per mouse per staining, as detailed below.

### Quantification of cortical cell density

To measure cortical cell density, sections taken from 2, 4, 6, and 10 month-old WT and TgMHu2ME199K/KO mice were evaluated. Eight microscopic images of Dapi-stained nuclei were obtained in a blinded manner from each brain section of the cortical hemispheres in × 20 magnification. Each mouse brain was evaluated in coronal sections at the level of Bregma = 0.00 ± 0.1 mm. Two adjacent fields midway between pial lining and corpus callosum at Bregma L = 1.0 mm were counted from each hemisphere (4 sections, 4 fields per section). Computerized quantification of nuclei was performed using ImageJ (Process 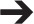 Binary 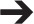 Watershed 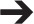 Analyze Particles), using a size-based threshold. An average value was calculated per animal, followed by calculation of the group (± SEM) average values. Nuclear counts for 2-months old wild-type mice were defined as baseline (100%) for comparison.

### Quantification of cortical neuronal and synaptic density

To measure neuronal cell density in the cortex, sections taken from 2- and 10- month old WT (*n* = 5) and TgMHu2ME199K/KO (*n* = 5–7) mice at Bregma = 0.00 ± 0.1 mm were stained for NeuN and counted manually in a blinded manner adhering to stereological principles. Two adjacent x20 fields midway between the pial lining and the corpus callosum at Bregma L = 1.0 mm were counted from each hemisphere (4 sections, 4 fields per section). Average count in 2-months old wild-type mice was considered as baseline of 100% for comparison. In order to estimate the synaptic density in the cortex, 2- and 10- months old WT and TgMHu2ME199K/KO mice at Bregma = 0.00 ± 0.1 mm were stained for synaptophysin. Two microscopic images were obtained at the same position as for Neun quantification, at a magnification of x40 and identical camera exposure from each hemisphere (4 sections, 4 fields per section). Computerized analysis was performed for the fraction of synaptophysin-stained area from total area of the image. Synaptic density in the cortex of 2-month old wild-type mice was regarded as baseline 100% for comparison. Both NeuN and synaptophysin were calculated as average values per mouse, followed by calculation of group average.

### Quantification of hippocampal cells

DAPI stained nuclei were counted manually in the CA1 and CA3 hippocampal regions in order to obtain a quantitative assessment of neuron number. Counting was performed on each hemisphere, on four sections per brain (8 fields per region, per brain). Average values were calculated for each mouse, followed by calculation of group average.

### Quantification of neurogenesis

For identifying proliferating brain cells (neurogenesis), WT and TgMHu2ME199K/KO mice of different age groups were injected intraperitoneally with bromodeoxyuridine (BrdU, Sigma-Aldrich, 50 μg/g body weight) for 7 consecutive days prior to sacrifice. Immunofluorescent staining for BrdU (rat α-BrdU, Serotec) was performed as previously described (Fainstein et al., 2013). The total number of BrdU-stained nuclei per 10 μm thick section residing within the anatomic region of the sub-ventricular / sub-ependymal zone (SVZ - SEZ) was counted manually at magnification of x20 at sections taken from Bregma = 0.00 ± 0.1 mm. For identifying hippocampal neurogenesis, BrdU-stained nuclei were counted manually in the sub-granular zone of the dentate gyrus of sections taken from Bregma = −2.0 mm. Average values from 4 sections were calculated for each mouse, followed by calculation of group average.

### Statistical analyses

TgMHu2ME199K/KO mice were compared to their ages-matched wild-type C57BL/6 control group using the nonparametric Mann-Whitney test.

## Results

### Progressive neurological impairment in E200K mice

**Figure 1:**
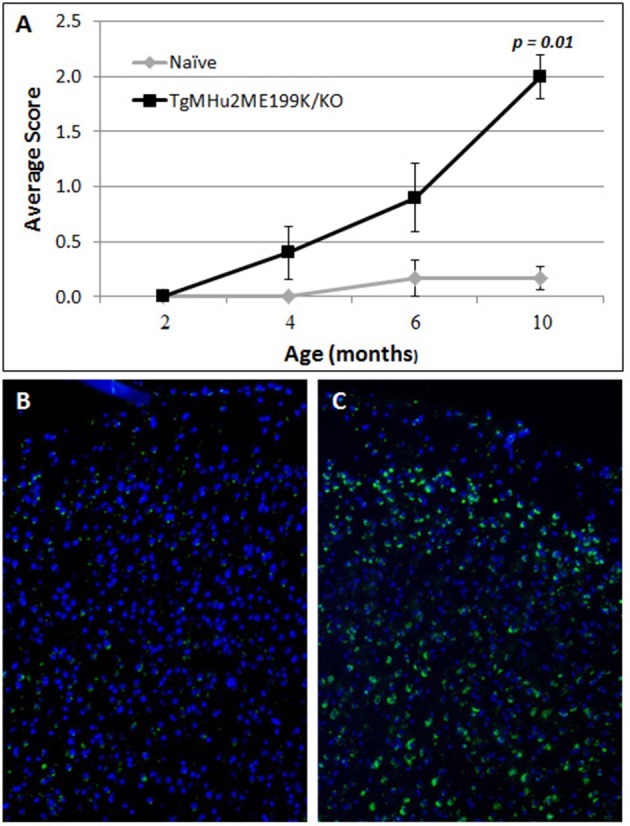
**Progressive neurological impairment in E200K mice**. TgMHu2ME199K/KO mice showed progressive neurological disability becoming apparent around age 6 month. Mice developed progressive hind leg weakness, as observed at age 10 months **(A)**. PrP^C^ aggregates are barely detected in the cortex of 2-month old TgMHu2ME199K/KO mice **(B)** and highly prevalent in the cortex of 10-month old TgMHu2ME199K/KO mice **(C)**.

TgMHu2ME199K/KO mice began to show hind limb weakness at age 4–6 months, followed by progressive neurological deterioration. At the age of 10 months mice exhibited significant (*p* = 0.001) hind limb weakness (Figure 1A). PrP^C^ aggregates, the disease hallmark, were marginally detected in the cortex of 2 month-old mice, while considerable load of aggregates was spotted at 10 months of age (Figures 1B,C).

### Age-related cortical neurodegeneration in E200K mice

**Figure 2:**
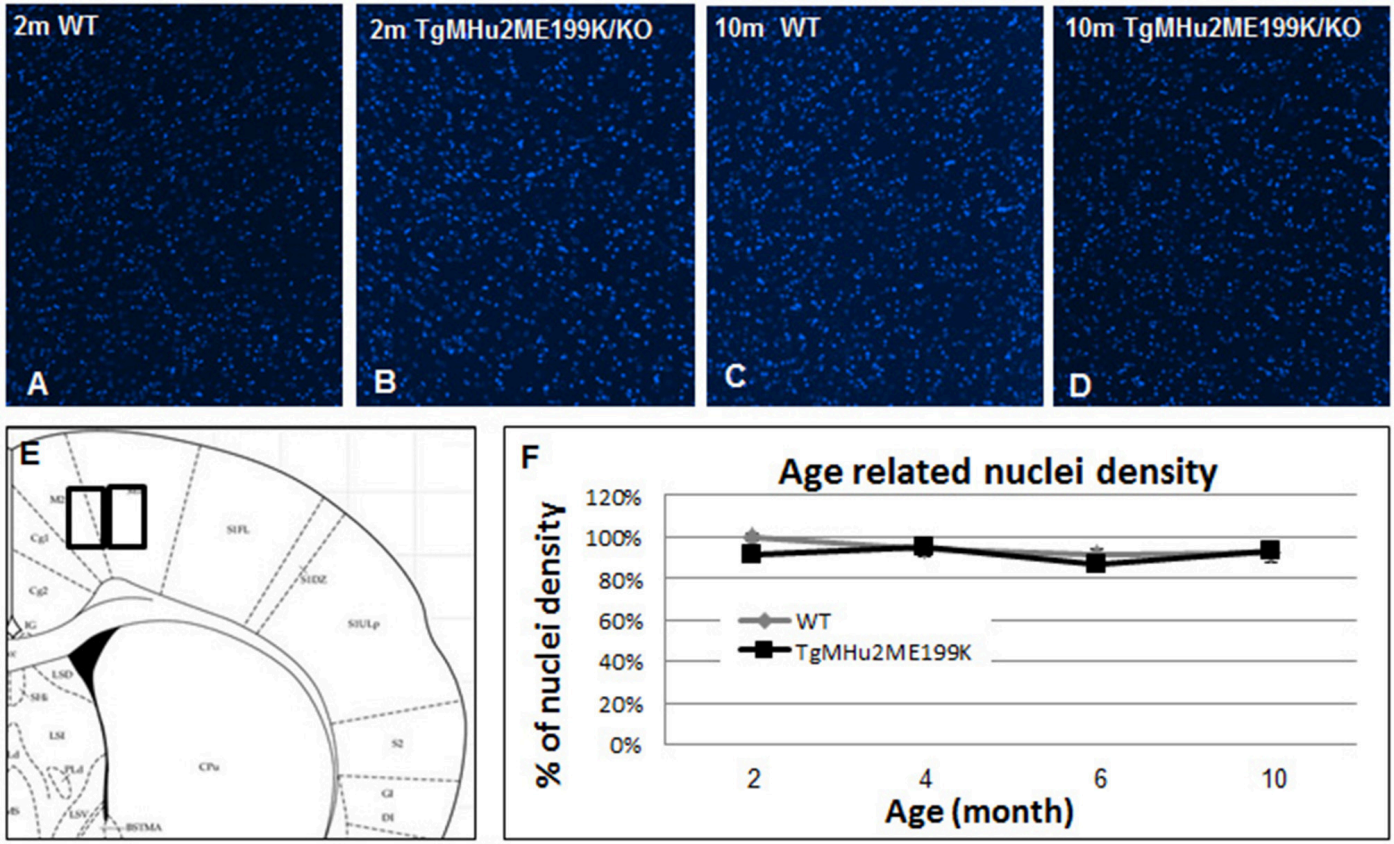
**Cortical cell density in TgMHu2ME199K/KO and wild-type mice**. Low power field images showing the overall nuclear density in the cortex of 2 month- (**A**- wild-type, **B**- TgMHu2ME199K/KO mice), and 10 month-old wild-type and TgMHu2ME199K/KO mice (**C**,**D**, respectively). Quantification of nuclear density was performed on high power field images according to the scheme shown in **(E)**. Quantification did not show age-related changes, and there was no difference between the two experimental groups throughout the follow-up period **(F)**.

In light of progressive neurological disability, we examined whether there is a neurodegenerative process in the cortex of TgMHu2ME199K/KO mice. First, we performed computerized quantification of cell density (by number of cell nuclei) in the cortex at ages 2, 4, 6, and 10 months. No difference in cortical cell density of TgMHu2ME199K/KO mice as compared to wild-type mice was detected (Figure 2).

**Figure 3:**
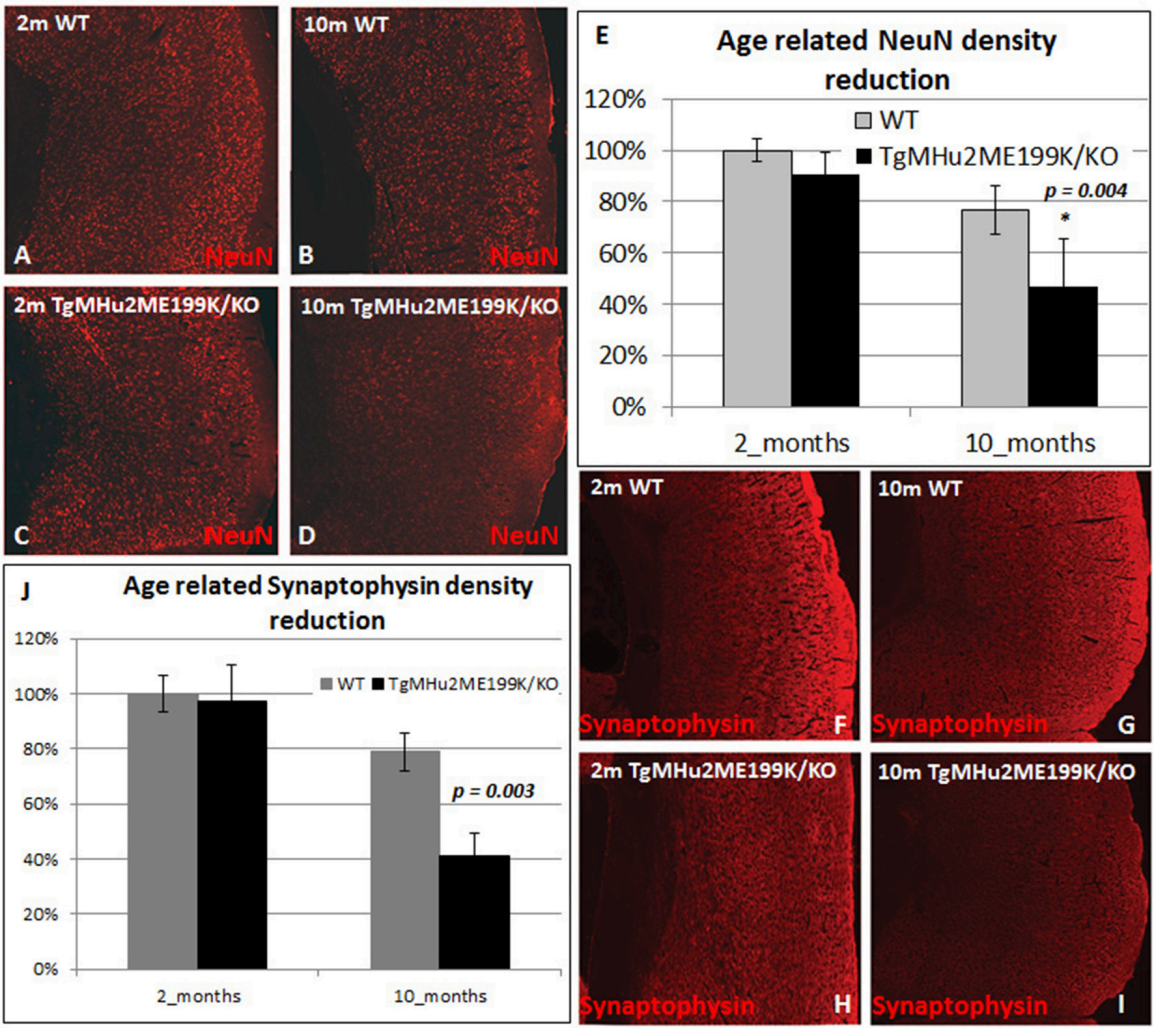
**Age-dependent cortical neuronal and synaptic loss in TgMHu2ME199K/KO mice**. Low power field images showing the overall density of cortical NeuN+ neurons **(A–D)** and Synaptophysin **(F–I)** in 2 month- and 10 month-old wild-type and TgMHu2ME199K/KO mice. Quantification was performed on high power field images according to the scheme shown in Figure 2E. No difference was observed in the number of NeuN+ cells between the groups at 2 month of age **(A,C,E)**. WT mice exhibited physiological, age related **(A,B,E)** 23.3% decrease in cortical NeuN+ cells (*p* = 0.015). TgMHu2ME199K/KO mice exhibited 53% (*p* = 0.046) reduction in cortical NeuN+ cells **(C–E)** at the age of 10 months. Analysis of the synaptic density, revealed similar findings. There was no difference between TgMHu2ME199K/KO and WT mice at 2 months of age. There was a physiological 21% decrease in synaptic density in WT mice at 10 months (*p* = 0.02, **F,G,J**) versus 59% **(H,I,J)** decrease in TgMHu2ME199K/KO mice (*p* = 0.004).

We therefore examined whether there is a selective degenerative process affecting predominantly cortical neurons. We quantified the density of NeuN+ neurons in the cortex (Figures 3A–E). At 10 months, there was a physiologic age-related 23.3% loss of cortical neurons in wild-type mice. In TgMHu2ME199K/KO mice, cortical neuron density was similar at 2 months, but declined by 48.5% at 10 months, significantly lower than wild-type mice (*p* = 0.04648). In view of the neuronal loss, we examined synaptic density by computerized analysis of synaptophysin immunostaining (Figures 3F–J). At age 2 months, there was no difference in synaptic density between TgMHu2ME199K/KO and wild-type mice. At 10 months, synaptic density declined by 25% in wild-type mice and by 59% in TgMHu2ME199K/KO mice (*p* = 0.004). Thus, synaptic density declines in E200K mice in a manner comparable to loss of neurons.

In conclusion, TgMHu2ME199K/KO mice exhibited cortical neurodegeneration.

### Age-related decline in neurogenesis in the sub-ventricular / sub-ependymal zone in TgMHu2ME199K/KO mice

**Figure 4:**
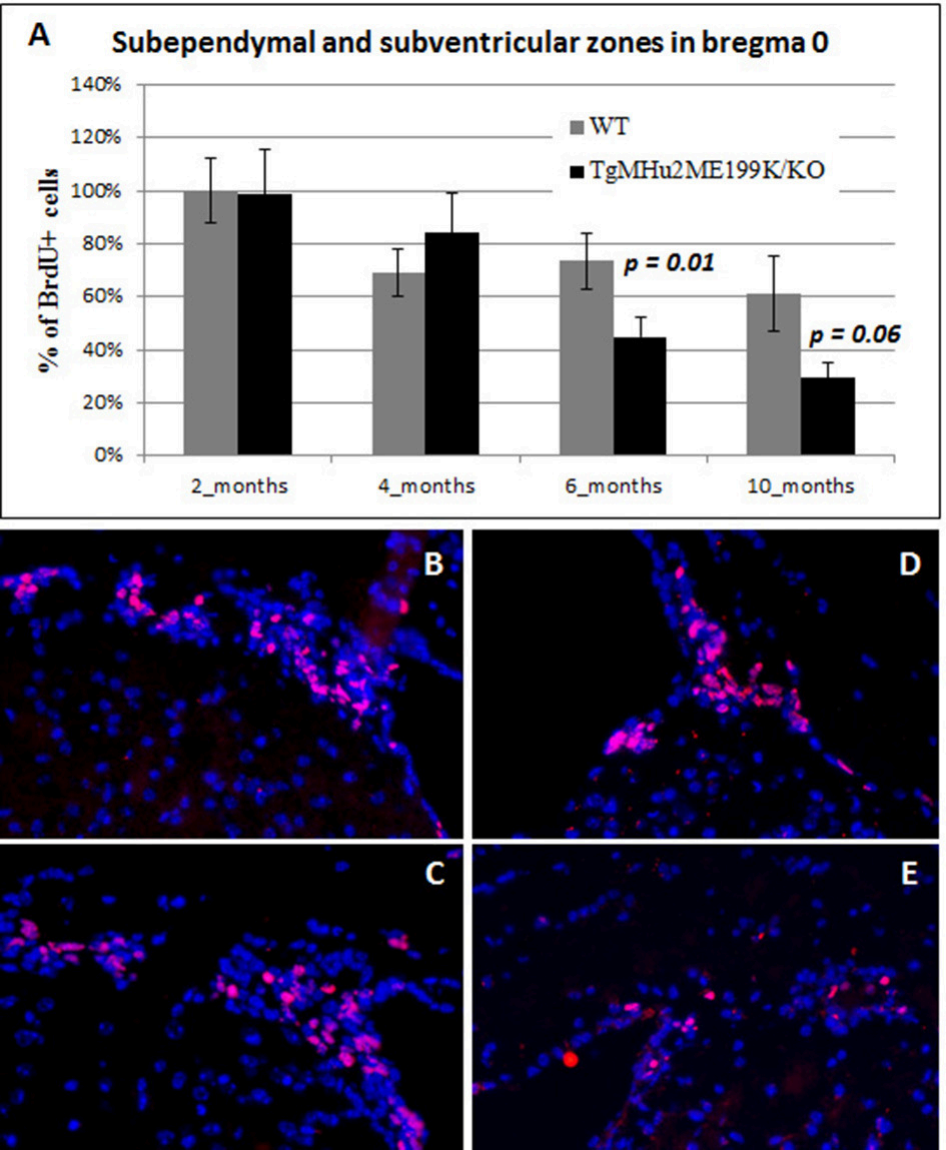
**Age related decline in neurogenesis in sub-ventricular / sub-ependymal zone in TgMHu2ME199K/KO mice**. BrdU incorporating cells were quantified in the sub-ventricular / sub-ependymal zone. Similar neurogenesis rates were detected in young 2- and 4-month old WT and TgMHu2ME199K/KO mice in the SVZ / SEZ. However, at 6 and 10 months of age, there was a significant reduction in neurogenesis in TgMHu2ME199K/KO versus WT mice (*P* = 0.01 and *P* = 0.06 respectively) in both areas **(A)**. Representative images show neurogenesis in 2 month-old wild-type and TgMHu2ME199K/KO mice **(B,C)**, and in 10 month-old mice **(D,E**, respectively).

We further examined the rate of neurogenesis in the sub-ventricular / sub-ependymal zone (SVZ - SEZ) of wild-type and TgMHu2ME199K/KO mice. Specifically, we quantified BrdU incorporation at the level of Bregma 0. There was an age-related physiological decline in neurogenesis in wild-type mice. The number of BrdU+ cells at 6 months showed a 27% decline and dropped further at 10 months, by 39% (*p* = 0.02 as compared to 2 months) respectively, from the baseline level (Figure 4A).

The rate of neurogenesis was comparable in TgMHu2ME199K/KO and wild-type mice at 2 months (Figures 4B,C). However, neurogenesis declined to a significantly lower rate of BrdU incorporation in TgMHu2ME199K/KO mice as compared to wild-type at 6 and 10 months (Figures 4D,E). The number of BrdU+ cells decreased by 40 and 76% at age 6 and 10 months respectively (*p* = 0.01 and *p* = 0.06 respectively). In conclusion, there is significant age-related decline in SVZ - SEZ neurogenesis as compared to physiologic ageing.

### Age-related memory impairment in TgMHu2ME199K/KO mice

**Figure 5:**
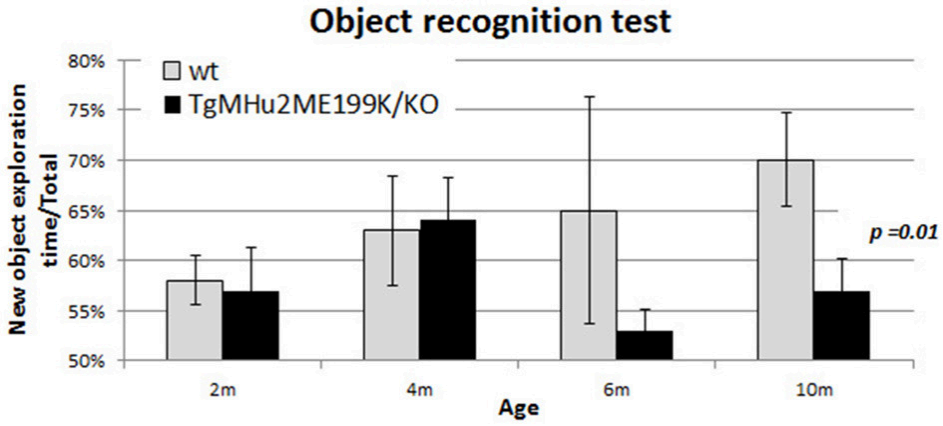
**Age-related memory impairment in TgMHu2ME199K/KO mice**. No difference was detected in the performance of 2 and 4 month-old TgMHu2ME199K/KO and WT mice in the object recognition test, a hippocampal-dependent memory assessment. However, at 6 months of age a trend (*p* = 0.06) toward poorer performance was noted in TgMHu2ME199K/KO mice. Ten month-old WT mice performed significantly better than TgMHu2ME199K/KO age-matched mice (*p* = 0.01).

In order to examine whether TgMHu2ME199K/KO transgenic mice also develop age-related memory impairment, they were examined at ages 2–10 months using the object recognition test (Bevins and Besheer, 2006; Figure 5). Their performance was compared to wild-type mice. There was no difference in performance at ages 2 and 4 months. A trend of decline was noted at 6 months of age, and at 10 months there was statistically-significant memory impairment in TgMHu2ME199K/KO mice (*p* = 0.01).

### Age-related degeneration of the hippocampus in TgMHu2ME199K/KO mice

**Figure 6:**
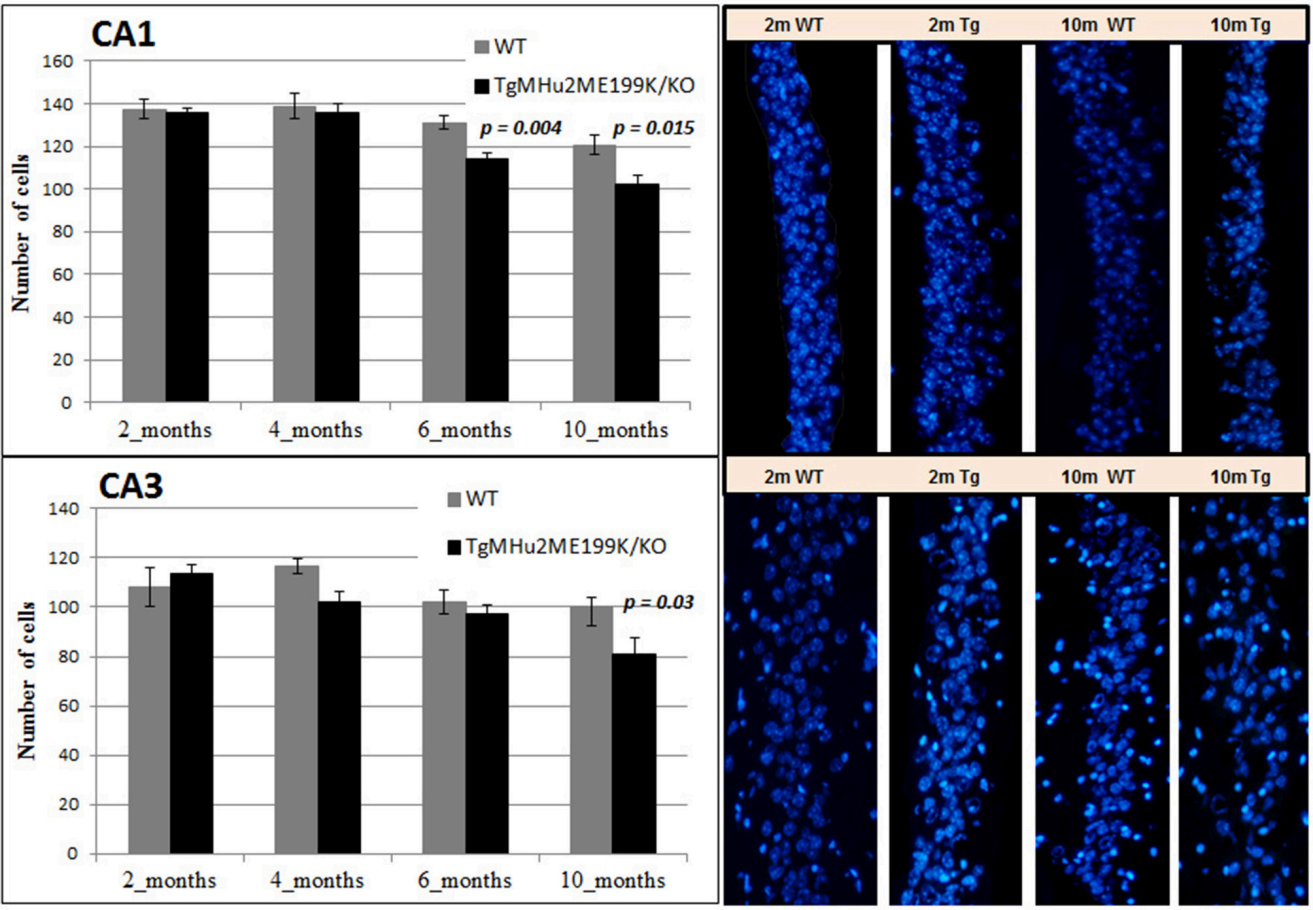
**Age-related degeneration of the hippocampus in TgMHu2ME199K/KO mice**. No difference was observed in the number of CA1 cells in the hippocampus of young 2 and 4 months WT and TgMHu2ME199K/KO mice (upper left panel). Progressive reduction in the number of CA1 cells was observed starting at 6 months of age and further at 10 months of age (Upper right panel, *p* = 0.004 and *p* = 0.015, respectively). In the CA3 hippocampal area (lower panel) there was a significant decline (*P* = 0.03) in cell number in TgMHu2ME199K/KO versus WT mice at 10 month of age.

We quantified neuronal cells in the CA1 and CA3 regions at several time points (Figure 6). In wild-type mice, peak counts of neurons were found at 4 months, with a mild decline at ages 6 and 10 months (Figure 6). At ages 2 and 4 months there was no difference in neuronal counts between TgMHu2ME199K/KO and wild-type mice. A significant 13% decrease in number of neurons was observed in the CA1 region of TgMHu2ME199K/KO mice as compared to wild-type at 6 months (*p* = 0.004), and 16% decline at 10 months (*p* = 0.015). In the CA3 region, a 19% decrease was measured at the age of 10 months with borderline significance (*p* = 0.03). Thus, TgMHu2ME199K/KO mice exhibit an accelerated loss of hippocampal neurons as compared to physiologic aging of wild-type mice.

### Increased hippocampal neurogenesis in E200K mice

**Figure 7:**
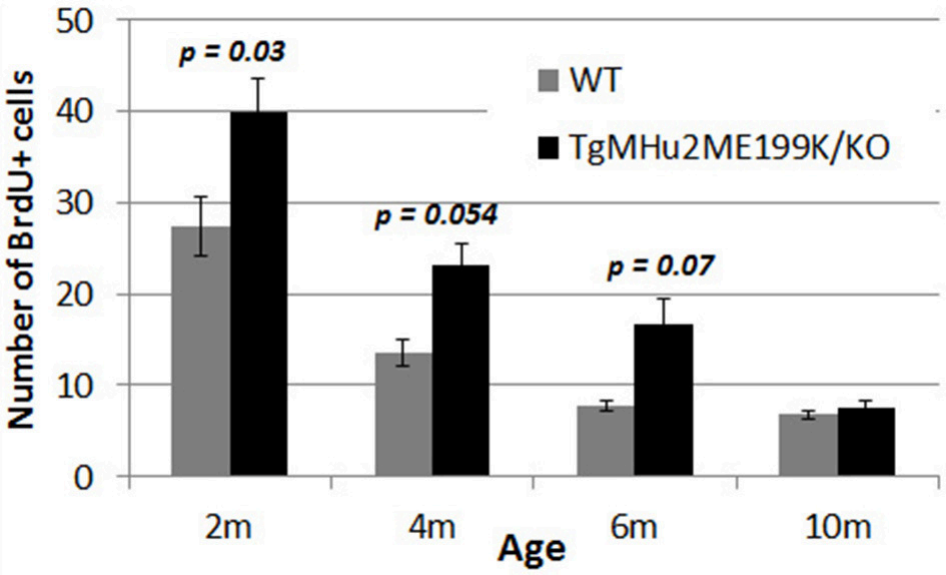
**Increased hippocampal neurogenesis in E200K mice**. There was progressive physiological decline in hippocampal neurogenesis with age. There was a higher number of BrdU incorporating cells in the subgranular zone of the dentate gyrus in TgMHu2ME199K/KO mice as compared to wild-type mice up to the age of 6 months (*p* = 0.07). At 10 months of age there was no difference in hippocampal neurogenesis between TgMHu2ME199K/KO and WT mice.

The generation of new neurons in the sub-granular zone of the dentate gyrus (Lennington et al., 2003) occurs throughout life, and plays an important role in memory functions (Snyder et al., 2005). We therefore examined the rate of neurogenesis in the dentate gyrus of TgMHu2ME199K/KO and wild-type mice at various ages. There was a physiologic decline in the rate of proliferation (indicated by uptake of BrdU) of subgranular-zone cells in wild-type mice (Figure 7). At age 2 months, there were 1.73-fold more BrdU+ cells in TgMHu2ME199K/KO mice, as compared to wild-type mice (*p* = 0.03). At ages 4 and 6 months there were 1.71- (*p* = 0.054) and 2.13 = (*p* = 0.07) fold more BrdU+ cells in the TgMHu2ME199K/KO mice as compared to wild-type. At age 10 months, neurogenesis had declined in both groups, by 75 and 85%, respectively, from that of 2 months, without significant difference between wild-type and TgMHu2ME199K/KO mice.

In conclusion, TgMHu2ME199K/KO mice exhibited memory impairment and loss of hippocampal volume as early as 6 months. These mice exhibited increased hippocampal neurogenesis initially, which declined thereafter and did not compensate for the neuronal death.

## Discussion

### A need for animal models of neurodegeneration

Neurodegenerative diseases represent one of the most important causes of morbidity and mortality in the aging population. The common feature of the various diseases is the progressive loss of brain neurons. To date, there are no available treatments to cure or even slow down the progression of neurodegeneration. Importantly, at the time of clinical diagnosis, there is already advanced and irreversible brain atrophy. Ideally, the optimal therapeutic approach would entail early, pre-symptomatic intervention with medications that provide wide protection of brain cells against the (common) pathogenic mechanisms that cause neurodegeneration. When addressing illness in the symptomatic stages, there is also a need for developing regenerative therapies. A major limitation in studying the mechanisms by which pathogenic events lead to brain atrophy and in developing neuroprotective and regenerative therapies is the critical shortage in animal models which display a neurodegenerative process. For example, this drawback is seen in most transgenic animal models of familial Alzheimer's disease, which exhibit typical pathologic features, including widespread deposition of β-amyloid plaques but without associated neuronal loss (Oddo et al., 2003a,b; Savonenko et al., 2005; Radde et al., 2006). Furthermore, memory impairment in the double-transgenic animal model of Alzheimer's disease is independent of hippocampal neuronal volume, and is completely reversed by inhibiting innate immune cytokines, namely interleukin-1(Ben-Menachem-Zidon et al., 2014), and TNFα (Tweedie et al., 2012). Even the commonly-used animal model of familial Alzheimer's disease, carrying a cassette of 5 mutated human genes (the 5XFAD mouse) that has been reported to express β-amyloid plaques as early as age 2 months and memory impairment at 4 months, exhibits only limited neuronal loss, observed mainly in cortical layer 5 and subiculum of the hippocampus at 9 months (Oakley et al., 2006). Although, local Aβ42 deposition was elegantly shown to correlate with apoptotic activity and neuronal loss in this brain region of 5XFAD mice (Eimer and Vassar, 2013), a major objective remains to develop animal models which display progressive and substantial loss of brain neurons which precedes cognitive decline.

### TgMHu2ME199K/KO mice serve as a model for neurodegenerative diseases

**Figure 8:**
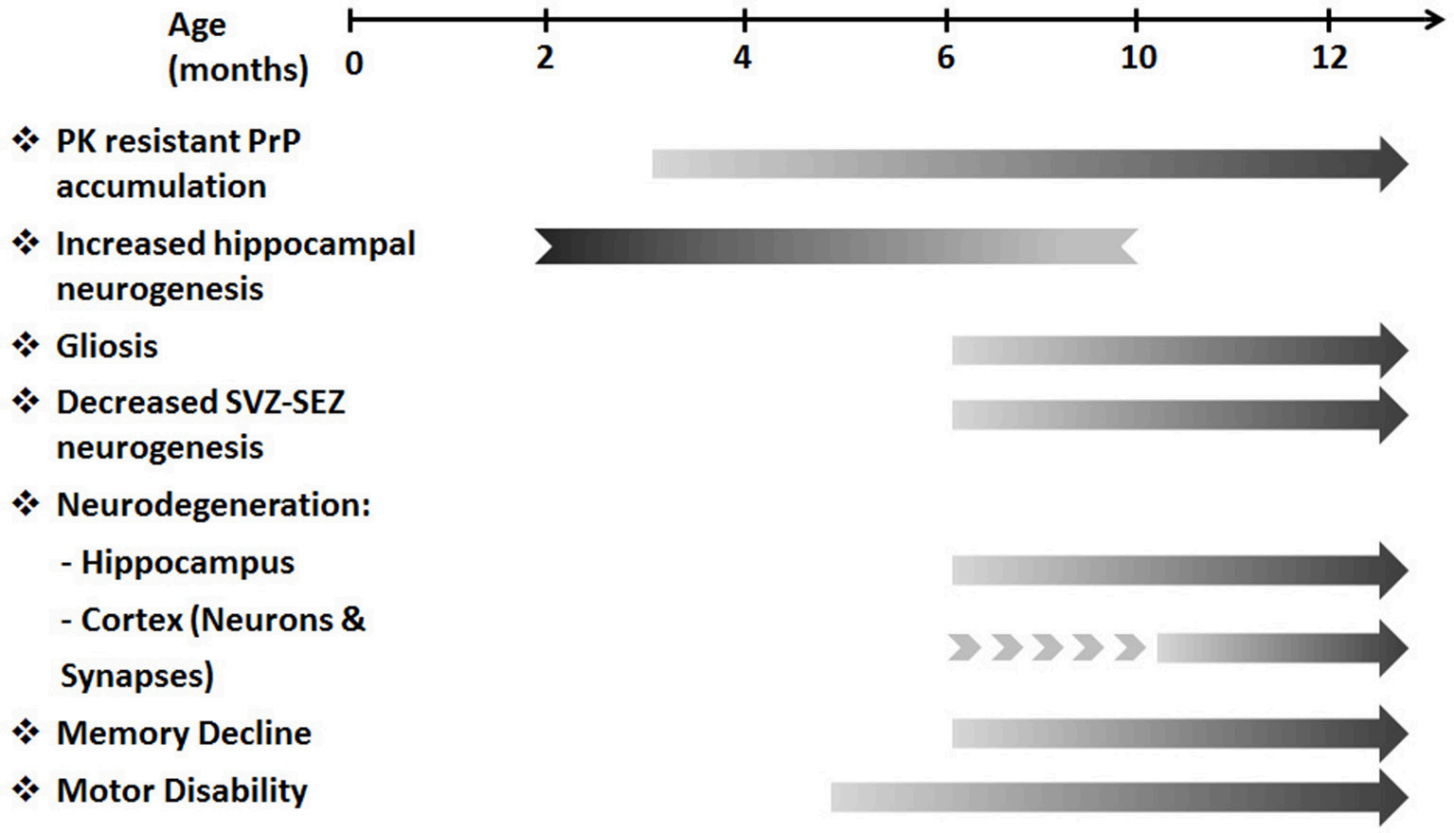
**A temporal flow chart of clinical and pathological manifestation in TgMHu2ME199K/KO model**. The pre-clinical stage of the disease starting from 3 months of age is characterized by PK resistant PrP accumulation. Subsequently, at 5–6 months of age first motor deficits are revealed. As disease progresses gliosis, cortical and hippocampal neuro-degeneration, decreased SVZ-SEZ neurogenesis and memory deficits are detected. In addition, increased hippocampal neurogenesis identified in young TgMHu2ME199K/KO mice diminishes with age. The clinical disability and the pathological aberrations continue to deteriorate with disease progression.

The overlapping features of prion diseases and other neurodegenerative diseases highlight their potential to serve as a model for studying certain aspects of neurodegeneration and neuroprotection. TgMHu2ME199K/KO mice serve as an animal model for genetic prion disease, carrying the mutated human gene for the prion protein. Given the progressive decline in motor performance in TgMHu2ME199K/KO mice, we performed longitudinal characterization of the pathologic features of brain atrophy in these mice as compared to wild-type mice. This study presents for the first time histopathological evidence for a neurodegenerative process in transgenic TgMHu2ME199K/KO mice, exhibiting progressive loss of cortical and hippocampal neurons. This started at a pre-clinical age of 4–6 months, followed by memory impairment, which started after 6 months. The longitudinal study revealed the dynamics of the neurodegenerative process, and indicated the existence of an accelerated process of neuronal loss as compared to normal physiologic aging in wild-type animals. Figure 8 summarizes the clinical and pathological features of this animal model. Importantly, it shows the temporal correlation between the neurodegenerative pathology and development of clinical impairments.

### Mechanisms of neurodegeneration which can be studied in TgMHu2ME199K/KO mice

Several mechanisms of neuronal injury are common to multiple neurodegenerative diseases. For example, the role of oxidative stress by reactive oxygen species (Van Everbroeck et al., 2004) and microglial toxicity (Brown, 2001; Amor et al., 2010) has been shown in prion diseases, as well as in Alzheimer's disease (Rapic et al., 2013; Heneka et al., 2015; Matsumura et al., 2015), Parkinson's disease (Aras et al., 2014; Borrajo et al., 2014; Kim et al., 2015) and Huntington's disease (Crotti et al., 2014; Rotblat et al., 2014). Specifically, early oxidative stress causing lipid peroxidation is linked to propagation of pathologic prion protein accumulation and to typical neuropathological changes in prion disease (Brazier et al., 2006). Similarly, oxidation of nucleic acids, proteins and lipids are an early pathologic change in Alzheimer's disease (Nunomura et al., 2006). Also, the accumulation of prion protein is associated with activation of microglia, leading to neurotoxic injury and gliosis (Brown, 2001; Riemer et al., 2009). Similarly, activated microglia are involved in the pathogenesis of Alzheimer's disease, and β-amyloid toxicity is strongly increased by the presence of microglia (Qin et al., 2002). Therefore, we suggest that the transgenic TgMHu2ME199K/KO model can serve as the basis for developing neuroprotective and regenerative therapies that target the common mechanisms of injury in neurodegenerative diseases. Indeed, our group has recently shown the protective properties of anti-oxidant therapy with nanoparticles of punicic acid emulsions in the TgMHu2ME199K/KO mouse (Mizrahi et al., 2014).

This study shows decreased neurogenesis in the subependymal / subventricular zone of TgMHu2ME199K/KO mice at age of 6 months. The prion protein is expressed in non-proliferating cells in the SVZ (Steele et al., 2006) and inhibits the differentiation of neural precursors (Relaño-Ginès et al., 2013). Interestingly, there was increased neurogenesis in the subgranular zone of the dentate gyrus up to age 6 months, and then a decline to the low level of neurogenesis in physiologically aging mice. These findings confirm the recent report on increased hippocampal neurogenesis in the infective form of prion disease (Gomez-Nicola et al., 2014). In the wider perspective of neurodegenerative diseases, animal models of familial Alzheimer's disease more often exhibit decreased hippocampal neurogenesis (Haughey et al., 2002; Rodríguez et al., 2008; Demars et al., 2010), although normal or even increased proliferative response to neurogenic stimuli has been observed (Mirochnic et al., 2009; Perry et al., 2012). Several studies of mouse lines representing familial Alzheimer disease, mostly overexpression of APP, showed increased hippocampal neurogenesis (Jin et al., 2004; Chuang, 2010). A pathological study of human Alzheimer's disease brain specimens found a reduced number of hippocampal stem cells with increased proliferation (Perry et al., 2012). Therefore, our findings in TgMHu2ME199K/KO mice may further represent other neurodegenerative diseases. There is a need to further characterize the dynamics of neurogenesis in prion diseases, and whether the inability of increased subgranular zone neurogenesis to compensate for hippocampal neuronal loss is the result of blocked differentiation or increased cell death.

In conclusion, transgenic TgMHu2ME199K/KO mice serve as a model of chronic neurodegenerative disease. This model can serve in studying the role of common disease mechanisms which promote neurodegeneration and in testing neuroprotective and pro-regenerative agents.

## Author contributions

NF: Conception and design, Collection and/or assembly of data, data analyses and Manuscript writing; DD: Preparation and provision of study material, Collection and/or assembly of data, Data analysis and interpretation; KF: Preparation and provision of study material; AF: Collection and/or assembly of data; IS: Preparation and provision of study materials; RG: Conception and design, manuscript preparation, final approval of the manuscript; TB: Conception and design, manuscript preparation, final approval of the manuscript.

### Conflict of interest statement

The authors declare that the research was conducted in the absence of any commercial or financial relationships that could be construed as a potential conflict of interest.
